# Flexible pressure sensors with ultrahigh stress tolerance enabled by periodic microslits

**DOI:** 10.1038/s41378-023-00639-4

**Published:** 2024-02-08

**Authors:** Song Wang, Chenying Wang, Yifan Zhao, Yujing Zhang, Yaxin Zhang, Xiangyue Xu, Qijing Lin, Kai Yao, Yuheng Wang, Feng Han, Yu Sun, Zhuangde Jiang

**Affiliations:** 1 State Key Laboratory for Manufacturing Systems Engineering, International Joint Laboratory for Micro/Nano Manufacturing and Measurement Technologies, School of Mechanical Engineering, Xi’an Jiaotong University, Xi’an, China; 2 State Key Laboratory for Manufacturing Systems Engineering, International Joint Laboratory for Micro/Nano Manufacturing and Measurement Technologies, School of Instrument Science and Technology, Xi’an Jiaotong University, Xi’an, China; 3 The Faculty of Electrical Engineering and Computer Science, Ningbo University, Ningbo, China; 4 Department of Mechanical and Industrial Engineering, University of Toronto, Toronto, Canada

**Keywords:** Sensors, Electrical and electronic engineering

## Abstract

Stress tolerance plays a vital role in ensuring the effectiveness of piezoresistive sensing films used in flexible pressure sensors. However, existing methods for enhancing stress tolerance employ dome-shaped, wrinkle-shaped, and pyramidal-shaped microstructures in intricate molding and demolding processes, which introduce significant fabrication challenges and limit the sensing performance. To address these shortcomings, this paper presents periodic microslits in a sensing film made of multiwalled carbon nanotubes and polydimethylsiloxane to realize ultrahigh stress tolerance with a theoretical maximum of 2.477 MPa and a sensitivity of 18.092 kPa^−1^. The periodic microslits permit extensive deformation under high pressure (*e.g*., 400 kPa) to widen the detection range. Moreover, the periodic microslits also enhance the sensitivity based on simultaneously exhibiting multiple synapses within the sensing interface and between the periodic sensing cells. The proposed solution is verified by experiments using sensors based on the microslit strategy for wind direction detection, robot movement sensing, and human health monitoring. In these experiments, vehicle load detection is achieved for ultrahigh pressure sensing under an ultrahigh pressure of over 400 kPa and a ratio of the contact area to the total area of 32.74%. The results indicate that the proposed microslit strategy can achieve ultrahigh stress tolerance while simplifying the fabrication complexity of preparing microstructure sensing films.

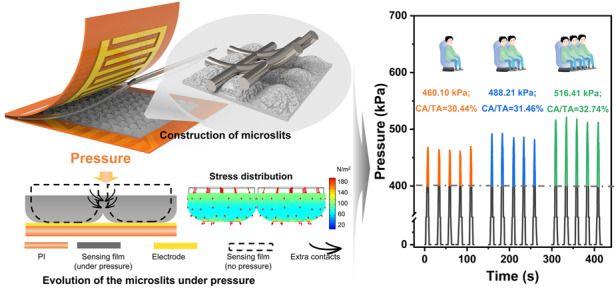

## Introduction

Significant progress has been made recently in the development of flexible pressure sensors that mimic the pressure-sensing capabilities of human skin and collect environmental data^1–3^. These advanced sensors have been widely applied in various fields, including human-computer interaction^4–9^, health monitoring^10–13^, and intelligent robotics^14,15^. Flexible piezoresistive pressure sensors have been widely used because of their simple manufacturing process, capability to record signals, and affordability. One of the common ways to achieve sensing films with excellent mechanical properties, stability, and durability is to incorporate conductive nanoparticles into an elastic matrix as a sensing material^16,17^. Typically, carbon-based nanomaterials, such as carbon black^18,19^, carbon fibers^8^, and graphene^20,21^, are conventionally used as conductive fillers. Additionally, carbon nanotubes (CNTs) offer excellent electrical properties, low density, and high elasticity, making them advantageous for sensors in terms of sensitivity, robustness, and stability compared to other materials^17,22–25^. Particularly, multiwalled carbon nanotubes (MW-CNT) have been considered highly promising due to their exceptional electromechanical performance and ease of preparation compared to single-walled carbon nanotubes^26^.

Recently, various strategies have been proposed for improving the stress tolerance of piezoresistive flexible pressure sensors and expanding their sensitivity, including pyramidal^27,28^, dome-shaped^29^, wrinkled^30^, and hierarchical configurations^31–34^. These innovative designs have shown promising application potential, but their fabrication process often involves complex molding and demolding processes with cumbersome coating and molecular self-assembly steps (SAMs)^35^.

For practical applications, pressure sensors should be flexible and sensitive and have a wide detection range. A common strategy for improving the stress tolerance of pressure sensors has been to fabricate continuous microstructures interfacially^36–39^. However, when an externally applied pressure is exerted, the force is concentrated at the root of the continuous microstructures^28,40,41^, which can lead to distortions within adjacent cells and limit the sensing range. An overview of the existing flexible pressure sensors with diverse microstructures and their fabrication techniques is presented in Table 1. Nevertheless, in the preparation of these sensors, complex molding and demolding processes are performed, and continuous microstructures limit the sensing performance. In this study, a reasonable prediction of a 2.477 MPa pressure tolerance is achieved for the contact area using the in situ observation method.

**Table 1:** Summary of different microstructures and their fabrication methods and pressure sensing performance

Ref.	Microstructure type	Preparation methods	Sensing range		
			Experimentally/Maximum (kPa)		Theoretical maximum (MPa)
^44^	Fingerprint-microstructure	Laser manufacturing & Magnetron sputtering & Dipping	0–4.5	4.5	–
^45^	Strip-shaped bulges	Demolding from leaves & Chemical vapor deposition & Film transfer and conformal covering	0–5.8	5.8	–
^41^	Multistage ridges	Demolding from leaves & Thermally evaporating	0–7	7	–
^33^	Tentacle-like conical micropillars	Laser-engraving & Scratch coating & Demolding	0–23	23	–
^32^	Micro Cone Array	Two-step demolding process from leaves & Spray-coating	0.2–25	25	–
^46^	Bionic hierarchical structure	Demolding from abrasive papers & Depositing	0–90	90	–
^4^	Microdome	Two-step demolding process from rose petal & Sputtering	0.058–98.7	98.7	–
^35^	Pollen-shaped	Two-step demolding process from pollen & Drop-casting	0–218	218	–
^47^	Hemispherical arrays and gradient pores	Four-step pressing & Demolding & Removing the sacrificial template	0–400	400	–
This work	Microslits	Screen printing	0–400	400	2.477

It is hypothesized that introducing sufficient space to permit extra deformation between continuous microstructural cells can help to enhance stress tolerance in a sensing film. To test this hypothesis, microslits are constructed in the sensing film during the screen printing process to achieve ultrahigh stress tolerance. A sensing film, MW-CNT/polydimethylsiloxane (PDMS), with a periodic microslit morphology was developed with an ultrahigh stress tolerance of 400 kPa experimentally and a theoretical maximum stress tolerance of 2.477 MPa, and a sensitivity of 18.092 kPa^−1^ is achieved. The microslits in the MW-CNT/PDMS sensing film permit extensive deformation without requiring molding and demouling processes, leading to a distortion of only 26.7% (contact area/total area, CA/TA) under a high pressure of 400 kPa. Moreover, optimal ratios of MW-CNT/PDMS ensure multiple contacts successively in the sensing film and between the periodic sensing cells under loading, which enhances the sensing performance. The developed devices are employed for monitoring wind directions under a minute pressure. They also show reliable performance in medium-pressure sensing applications, such as robot movement and human health monitoring. Importantly, a vehicle load check is achieved for ultrahigh pressure sensing under an ultrahigh pressure of over 400 kPa and a maximal CA/TA ratio of 32.74%. The facile fabrication process of screen printing coupled with the proposed innovative microslit design has significant application potential in guiding the development of flexible devices that can withstand high-level stress.

## Results and discussion

**Fig. 1:**
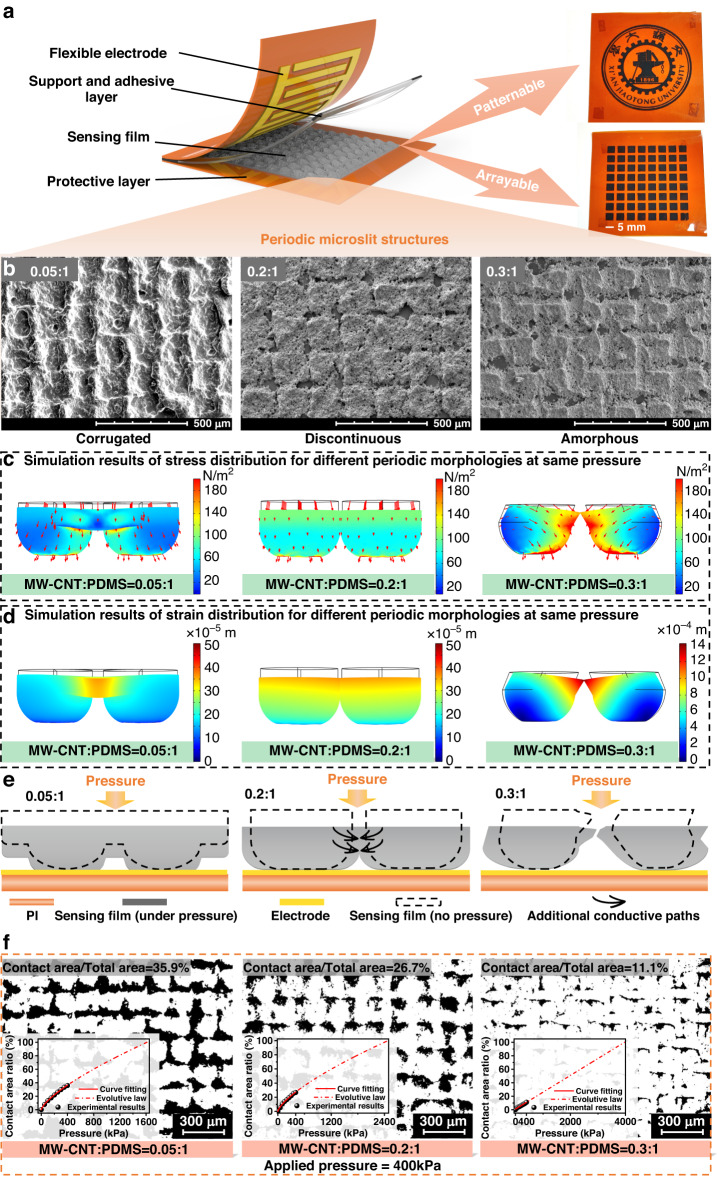
The sensor structure and characterization of the sensing film. **a** Structural diagram of the sensor facilitating patterning and array construction; **b** SEM images of the NW-CNT/PDMS sensing film with different weight ratios; the scale of all SEM images is 500 μm; **c** the simulated models of stress; **d** the simulated models of strain distributions; **e** schematic evolution in a distinct NW-CNT/PDMS sensing film; **f** the ratio of the contact area to the total area in the in situ measurement under an applied pressure of 400 kPa; the dark region represents the contact area, and the insets show the variations in the ratio with the applied pressure changing from zero to 400 kPa

The structure of the proposed sensor is presented in Fig. 1a. The sensor comprises flexible electrodes, support and bonding layers, a sensing film, and a protective layer. The sensing films were prepared employing screen printing technology using a mixture of MW-CNT and PDMS (refer to Fig. S1). The approach allows for straightforward patterning and arraying of sensing films, as illustrated in the right insets in Fig. 1a. Similarly, the Raman spectra of a well-blended film of PDMS and MW-CNT are presented in Fig. S2. Figure S3 shows that the film is lightweight and bendable and can be attached to bent fingers and leaf surfaces. The scanning electron microscopy (SEM) images in Fig. 1b and S4 indicate the presence of microslits in the MW-CNT/PDMS sensing films and show that microslits have been manifested successfully. In this study, various distinctive periodic microslit morphologies were observed by introducing microslits into sensing films with different MW-CNT/PDMS weight ratios. These morphologies were observed under the combined influence of the flowability of the complexes and the mold action of the screen-printed plates. In particular, the widths of the microslits of the sensing films increased with the MW-CNT/PDMS ratio. In addition, the introduction of the optimal microslits morphology led to a discontinuous square shape of the sensing morphology, with a ratio of 0.2:1. In contrast, as the ratio decreased, the morphology of the sensing film exhibited a continuous corrugation (a ratio of 0.05:1) without microslits; in contrast, when the ratio increased, it exhibited an unformed morphology (a ratio of 0.3:1) with widest microslits. Based on the results, the synergistic effect of the microslits and MW-CNT/PDMS is concluded to dominate the transition of the sensing morphology. To understand the mechanisms underlying the superior stress tolerance and sensitivity achieved in the film with a well-designed periodic microslits structure, twofold analysis was conducted. First, it is considered that the microslits configuration enables an extensive deformation to widen the detection range; second, it is considered that microslits synergize with the hierarchical microstructure to amplify the contact area during compression, which results in enhanced sensitivity.

**Fig. 2:**
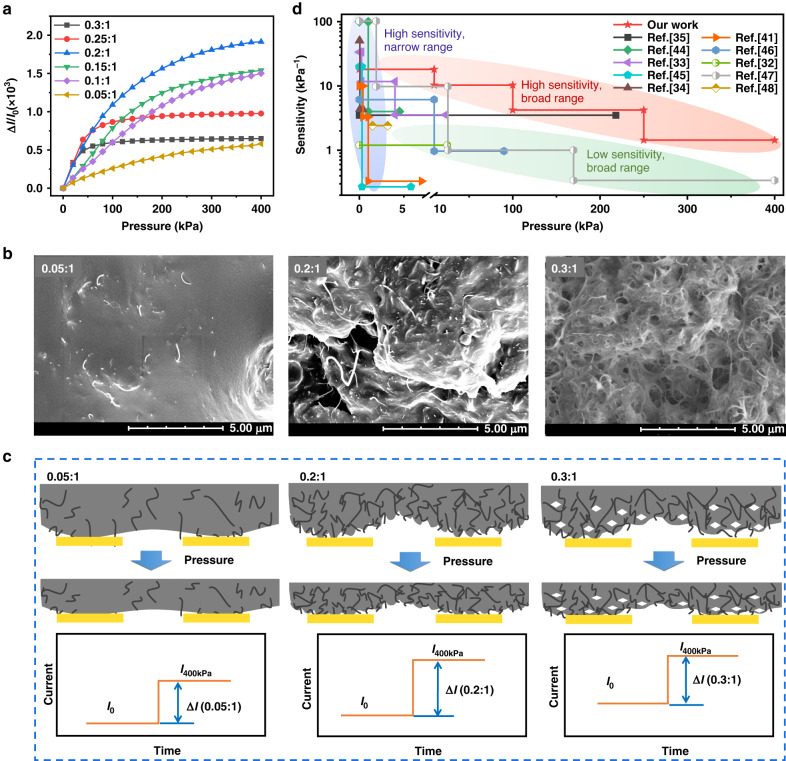
Properties and micromorphology of the MW-CNT and PDMS films. **a** The ${\boldsymbol{S}}$ curve of sensors for different weight ratios in the MW-CNT/PDMS films; **b** the microscopic morphologies of sensing films MW-CNT and PDMS with weight ratios of 0.05:1, 0.2:1, and 0.3:1; all the scales of SEM images are set to 5 μm; **c** the schematic microscopic morphologies corresponding to the current variations of sensing films with and without applied pressure; **d** the comparison results of the ${\boldsymbol{S}}$ curve and pressure sensing range of the reported flexible microstructure piezoresistive sensors^35,44–48^

The proposed sensing mechanism was proven as follows. First, finite element modeling (described in the Supporting Information) was used to simulate mechanical models for stress and strain distributions for different types of periodic microstructure morphologies observed in the SEM images, as shown in Fig. 1c, d. When pressure was applied, the adjacent corrugations in the MW-CNT/PDMS (0.05:1) sensing film without microslits were squeezed compactly, thus resulting in nonhomogeneous distributions of mechanical properties. The phenomenon that the stress and strain distortion could occur at the root contact area demonstrated that the forces and deformations were not adequately released because of limited available space between the adjacent periodic structures under pressure. The same tendency of nonhomogeneous distributions was observed in the MW-CNT/PDMS (0.3:1) sensing film with the largest microslits, but it exhibited larger variations in forces and deformations compared to the MW-CNT/PDMS (0.05:1) sensing film. This occurred because that amorphous morphologies originated from the collapse of the quadrate structure, causing an unbalanced distribution of stress and strain. However, symmetrical and balanced distributions of stress and strain occurred in the well-designed sensing film with MW-CNT/PDMS (0.2:1). This could be attributed to the presence of well-designed microslits that helped maintain the stability of periodic square structures. These suitable microslits could not only enable the homogeneous distribution of stress and strain throughout the film but also generate additional conductive paths (multiple synapses) when subjected to pressure, as shown in the evolution process illustrated in Fig. 1e. This further proved that the well-designed microslits could ensure superior sensing performance while enhancing stress tolerance. Furthermore, the in situ setup method (Fig. S5) with a maximal pressure of 400 kPa was employed for monitoring the stress tolerance of the sensing film under pressure to validate the simulation results. The endurance of applied pressure in the sensing film was quantified using the ratio of the contact area (CA under pressure) to the total area (TA tested area) of the sensing film, as shown in Fig. 1f. Ratios of 35.9% and 26.7% were detected in the MW-CNT/PDMS (0.05:1) and MW-CNT/PDMS (0.2:1) sensing films under a pressure of 400 kPa, respectively. This indicated that the MW-CNT/PDMS (0.2:1) sensing film with well-designed microslits had smaller deformation compared to the MW-CNT/PDMS (0.05:1) sensing film, reflecting its greater ability to endure higher pressure (maximum 2.477 MPa). Generally, the MW-CNT/PDMS (0.3:1) film exhibited wider microslits due to its unstable and irregular morphology. This characteristic allowed greater accommodation of microstructure deformation, resulting in enhanced resistance to the applied pressure. Consequently, the MW-CNT/PDMS (0.3:1) sensing film showed the lowest ratio of 11.1% under an applied pressure of 400 kPa. However, it should be noted that wider gaps between periodic cells of the MW-CNT/PDMS (0.3:1) sensing film impeded signal sensing, as shown in Fig. 2a. Therefore, the results provided strong experimental evidence that the MW-CNT/PDMS (0.2:1) sensing film could endure stress force and strain deformation, ensuring optimal sensing performance.

Next, the sensitive performance of the MW-CNT/PDMS sensing film was evaluated. The sensitivity performance of different sensor devices with different periodic morphologies is presented in Fig. 2a. The sensitivity (***S***) of the sensor device was calculated as:

1 $${\boldsymbol{S}}=\frac{\Delta {\boldsymbol{I}}/{{\boldsymbol{I}}}_{{\bf{0}}}}{\Delta {\boldsymbol{P}}}$$

where $\Delta {\boldsymbol{I}}$ represents the difference between the current value under the applied pressure (${\boldsymbol{I}}$) and the initial value without applied pressure (${{\boldsymbol{I}}}_{{\bf{0}}}$), and it is calculated as $\Delta {\boldsymbol{I}}{\boldsymbol{=}}{{\boldsymbol{I}}}_{{\boldsymbol{0}}}{\boldsymbol{-}}{\boldsymbol{I}}$; $\Delta {\boldsymbol{P}}$ is the pressure applied to the sensor.

Thus, the shift in the current is significant for ${\boldsymbol{S}}$. The current-voltage (${\boldsymbol{I}}{\boldsymbol{-}}{\boldsymbol{V}}$) curves of the sensor presented in Fig. S6 show linear variations, indicating an ohmic contact between the sensing film and the electrodes. This linear behavior indicated that the micromorphologies of the contact resistance had a significant effect on the sensing performance. To further demonstrate the impact of the well-designed microslits on sensitivity, Figure S7 presents the sensing mechanism of the microslits pressure sensor. The initial resistance of the sensor when uncompressed was calculated as:

2 $${\boldsymbol{R}}={\boldsymbol{R}}_{\text{a}}+{\boldsymbol{R}}_{\text{b}}+{\boldsymbol{R}}_{\text{c}}+{\boldsymbol{R}}_{\text{d}}$$

where ${{\boldsymbol{R}}}_{{\bf{a}}}$, ${{\boldsymbol{R}}}_{{\bf{b}}}$, ${{\boldsymbol{R}}}_{{\bf{c}}}$, and ${{\boldsymbol{R}}}_{{\bf{d}}}$ represent the resistance of electrodes, the contact resistance between the sensing film and electrodes, the bulk resistance of a sensing film, and the contact resistance between the periodic sensing cells, respectively.

When the sensor was subjected to external pressure, the microstructure of the sensing film was compressed and deformed, increasing the contact area between the sensing film and the electrodes and that between the periodic sensing cells, as shown in Fig. 1c, d; the evolution process is illustrated in Fig. 1e. Furthermore, the values of ${{\boldsymbol{R}}}_{{\bf{b}}}$ and ${{\boldsymbol{R}}}_{{\bf{d}}}$ decrease with the contact area, so these values denote critical factors affecting the sensor sensitivity. However, the variations in the ${{\boldsymbol{R}}}_{{\bf{a}}}$ and ${{\boldsymbol{R}}}_{{\bf{c}}}$ values were considerably smaller than those of ${{\boldsymbol{R}}}_{{\bf{b}}}$ and ${{\boldsymbol{R}}}_{{\bf{d}}}$, which indicated that these terms could be neglected. Therefore, the sensitivity of the sensor was obtained as:

3 $${\boldsymbol{S}}=\frac{\frac{{\boldsymbol{I}}-{{\boldsymbol{I}}}_{{\bf{0}}}}{{{\boldsymbol{I}}}_{{\bf{0}}}}}{\Delta {\boldsymbol{P}}}=\frac{\frac{{\boldsymbol{I}}}{{{\boldsymbol{I}}}_{{\bf{0}}}}-1}{\Delta {\boldsymbol{P}}}=\frac{\frac{{\boldsymbol{U}}}{{\boldsymbol{R}}}/\frac{{\boldsymbol{U}}}{{{\boldsymbol{R}}}_{{\bf{0}}}}-1}{\Delta {\boldsymbol{P}}}=\frac{\frac{{{\boldsymbol{R}}}_{{\bf{0}}}}{{\boldsymbol{R}}}-1}{\Delta {\boldsymbol{P}}}=\frac{\frac{{{\boldsymbol{R}}}_{{\bf{b}}{\bf{0}}}+{{\boldsymbol{R}}}_{{\bf{d}}{\bf{0}}}}{{{\boldsymbol{R}}}_{{\bf{b}}}+{{\boldsymbol{R}}}_{{\bf{d}}}}-1}{\Delta {\boldsymbol{P}}}$$

where ${\boldsymbol{U}}$ represents the bias voltage; ${\boldsymbol{R}}$ and ${{\boldsymbol{R}}}_{{\bf{0}}}$ denote the resistance of a sensing film under applied pressure and its initial value without any pressure applied; and ${{\boldsymbol{R}}}_{{\bf{b}}{\bf{0}}}$ and ${{\boldsymbol{R}}}_{{\bf{d}}{\bf{0}}}$ represent the initial values of resistances ${{\boldsymbol{R}}}_{{\bf{b}}}$ and ${{\boldsymbol{R}}}_{{\bf{d}}}$, respectively.

From this equation, the sensitivity was positively correlated with the variations in the contact area between the sensing film and electrodes and that between the periodic sensing cells.

As shown in Fig. 2a, the MW-CNT/PDMS sensing film (0.2:1) exhibited the best sensitivity over the entire range (0–400 kPa) among all of the films. This enhanced sensitivity is attributed to the well-designed microslits that caused variations in the ${{\boldsymbol{R}}}_{{\bf{d}}}$ value and the hierarchical microstructure morphology in each periodic structural cell, leading to changes in the ${{\boldsymbol{R}}}_{{\bf{b}}}$ value, as shown in the SEM images in Fig. 2b. As presented in Fig. 2b, when the MW-CNT were doped into the PDMS in infinitesimal amounts, a smooth surface of the MW-CNT/PDMS films (0.05:1) with several carbon nanotubes was observed. However, the discrepancies became even more noticeable as the doping amounts of MW-CNT increased. The intricate hierarchical morphologies could be obtained in both MW-CNT/PDMS films (0.2:1 and 0.3:1) so that the new contacts in the interface of the sensing film could be reproduced abundantly under the applied pressure. This resulted in the lowest ${\boldsymbol{S}}$ value in the MW-CNT/PDMS films (0.05:1) because the nearly smooth surface and the continuous structure without microslits, shown in Fig. 1b, could not afford extra contacts under the applied pressure. The MW-CNT/PDMS films (0.3:1) were expected to obtain the highest ${\boldsymbol{S}}$ values of all the tested films because of their complex and compact substructures; however, an unexpected consequence of ${\boldsymbol{S}}$ arose to hinder the performance of these films. As illustrated in Fig. 2b, multiple holes were observed in the SEM image of the MW-CNT/PDMS films (0.3:1), and these holes in the sensing film increased the sensor resistance under ${\boldsymbol{P}}$*.* The exceptionally wide microslits posed a challenge in establishing a cell-to-cell contact. These factors both limited the output piezoresistive signals. Moreover, the massive introduction of the MW-CNT resulted in the highest ${{\boldsymbol{I}}}_{{\bf{0}}}$ in the MW-CNT/PDMS films (0.3:1), as displayed in Fig. 2c, which also limited sensitivity. In contrast, the SEM images of the MW-CNT/PDMS sensing film (0.2:1) showed that multiple holes were almost evaded and prevented. Finally, the highest $\Delta {\boldsymbol{I}}$ in the MW-CNT/PDMS (0.2:1) confirmed that these films demonstrated the best sensing performance, which aligns with the trends of the curves as functions of ${\boldsymbol{S}}$ shown in Fig. 2a. The results in Fig. 2d demonstrate that the proposed sensor design was superior regarding the sensitivity and detection range to the other recently reported design. The outstanding performance of the proposed sensor design can be attributed to the introduction of suitable microslits and hierarchical submorphologies. These design features can effectively mitigate the excess forces and deformations while constructing the extra conducting paths.

**Fig. 3:**
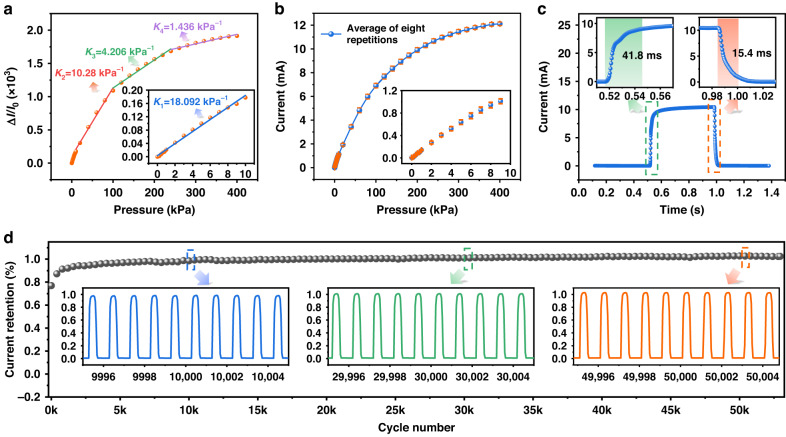
The performance of the proposed sensor with the MW-CNT/PDMS (0.2:1) sensing film. **a** Sensitivity of the sensor with the sensing film featured by MW-CNT/PDMS (0.2:1) with different intervals; **b** the average output current ${\boldsymbol{I}}$ of the sensor during eight cycles in full-scale loading of ${\boldsymbol{P}}$; the inset graph shows the standard deviation during eight cycles; **c** response and recovery time of the fabricated sensor; **d** the signal recording results of the sensor in 50,000 cycles with loading and unloading of 150 kPa

To explore potential applications of the sensor with periodic features in a sensing film, the ${\boldsymbol{S}}$ value was investigated within the pressure range of 0–400 kPa, as shown in Fig. 3a. The maximal ${\boldsymbol{S}}$ value of the sensor with the well-designed MW-CNT/PDMS (0.2:1) sensing film in different pressure intervals could be fitted to approximately 18.092 kPa^−1^ in the pressure range 0–10 kPa. As shown in Fig. 3a, the sensitivity slightly dropped to 10.28 kPa^−1^; additionally, a constant ${\boldsymbol{S}}$ value of 4.206 kPa^−1^ was exhibited when the applied pressure varied from 100 kPa to 250 kPa. Eventually, the sensitivity changed to 1.436 kPa^−1^ in the pressure range of 250 kPa–400 kPa. These results validated that the proposed sensor design is suitable for a variety of practical situations with different ${\boldsymbol{P}}$ values. Furthermore, the variations in the ${\boldsymbol{I}}$ value in the eight cycles with ${\boldsymbol{P}}$ from zero to 400 kPa are presented in Fig. 3b. The error bars in the inset graph in Fig. 3b represent the standard deviation of the measurement, which shows that the results of the eight cycles were uniform enough to achieve excellent robustness in sensing, which could be attributed to the superior stress tolerance of the squared periodic microslits in the MW-CNT/PDMS (0.2:1) film. Similarly, Fig. 3c presents the response and recovery time of the proposed sensor for the loading and unloading cases under an applied pressure of 150 kPa. The square step-shape response in the graph indicates an extremely fast response time of 41.8 ms and a recovery time of 15.4 ms. Figure 3d illustrates the remarkable stability of the sensor’s current ${\boldsymbol{I}}$ across more than 50,000 loading and unloading cycles under an applied pressure of 150 kPa, highlighting excellent stress tolerance and stability. The proposed microslit flexible pressure sensor exhibits hysteresis in its electrical response, as depicted in Fig. S8. This hysteresis is attributed to the viscoelastic nature of the MW-CNT and PDMS complex^42,43^. The mixture film cannot immediately return to its original state after pressure is released, resulting in relatively low current retention in the initial state, as shown in Fig. 3d. Moreover, the responses of the sensor under different frequencies of 0.5 Hz, 1 Hz, 2 Hz, and 4 Hz at a pressure of 250 kPa are displayed in Fig. S9, where favorable consistency can be observed, which further reinforced the reliability and suitability of the proposed sensor for particular applications. Furthermore, as shown in Fig. S10, an 8 × 8 sensor array with a resolution of 6.5 mm was tested to identify the magnitude and distribution of the pressure when the letters Y, E, and S were applied. These results demonstrated the good application potential of the well-designed sensor in sensing monitoring over a large area. Furthermore, the results in Fig. S11 indicate the current response of the sensor over the temperature range from zero to 40°C. The proposed sensor exhibited a small sensing variation and was applicable in most of the daily scenarios with respect to the varied temperatures. Then, the output currents of three samples of different batches were tested to verify the homogeneity. The calculated mean and standard deviation are shown in Fig. S12, which demonstrate good uniformity of the performance.

**Fig. 4:**
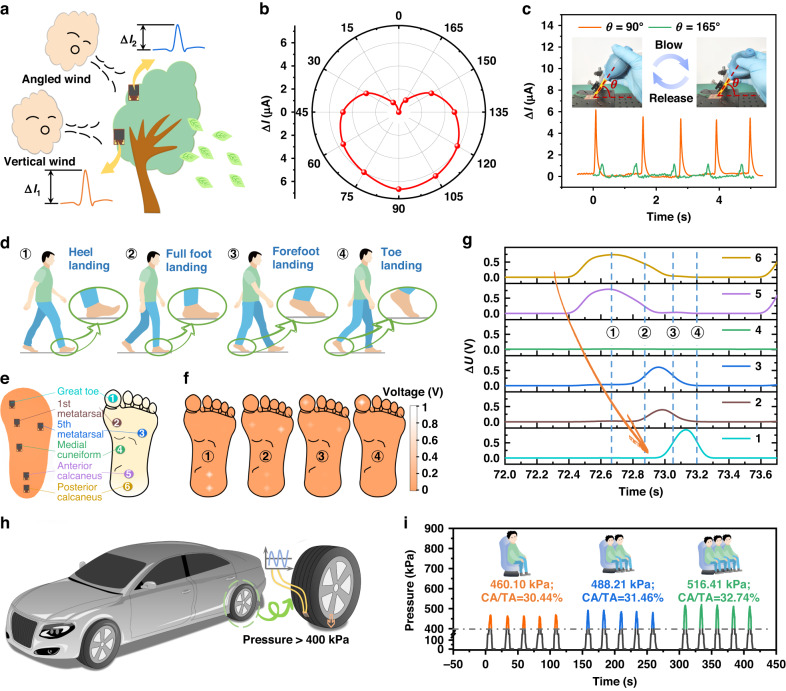
Practical applications of the proposed sensor. **a** The schematic diagram of the sensor applied to noncontact wind monitoring; **b** the relationship between the changes in signals and angles of the applied airflow; **c** the output response at the airflow of ${\boldsymbol{\theta }}$ = 90° and ${\boldsymbol{\theta }}$ = 165° in five cycles; the insets show the detailed experimental setup; **d** the sensor applied to human health monitoring: four typical events that occur during human locomotion can be accurately identified: ① heel landing, ② full foot landing, ③ forefoot landing, and ④ toe landing; **e** the schematic diagram of the smart insole arranged according to the key pressure monitoring locations on the bottom of the human foot; **f** the output distribution clouds of the sensors corresponding to the four events described in (**d**); **g** variations in the output of each sensor on the smart insole arranged on the right foot throughout locomotion; **h** the schematic diagram of vehicle load detection; **i** stable and multiple response signals of the proposed sensor under ultrahigh applied pressure of over 400 kPa

Figure 4a shows a noncontact sensing application based on the sensing performances achieved using the proposed periodic microslits design in the MW-CNT/PDMS film. In this application, a sensor was fixed on the leaves that could be distorted arbitrarily in a windy atmosphere. This design represents a feasible approach to quantitatively analyze the ambient wind direction due to the introduction of the microslit design in the sensing film, leading to excellent deformation capacity. Hence, the ambient airflow was experimentally imitated, and a schematic of the wind direction detection process is shown in Fig. 4c. The airflow angles (${\boldsymbol{\theta }}$) applied to the sensor could be switched from zero to 180° to follow the realistic atmosphere. Furthermore, as presented in Fig. 4b, when the airflow angles were horizontally shifted from zero and 180° to the vertical (90°) direction, the output of the sensor showed an increasing trend, achieving the maximum signal recording in the vertical direction. The heart-shaped sensing loop depicts the distinct variation to differentiate the directions of airflows. Figure 4c also demonstrates the stability and repeatability of wind direction detection at ${\boldsymbol{\theta }}$ **values** of 90° and 165°, respectively. Additionally, Figure S13 shows that the sensor output current increases with the airflow strength, indicating its suitability for monitoring wind magnitude. Therefore, by combining multiple sensors, it is possible to simultaneously monitor both wind strength and direction. These results validate that the presence of periodic microslit structures in a sensing film can support noncontact sensor application by outputting recognizable tiny signals. Furthermore, the designed sensor also demonstrates its capability in detecting robot finger joint bending, grasping objects, walking postures, and pulses for conventional contact applications, as shown in Figs. S14 and S15. Considering the deformation of the sensor in several application scenarios, it is crucial to explore the shift in sensing performance before and after repeated deformation. From Fig. S16, the favorable sensor performance can be maintained by bending over 1000 cycles. This indicates that our strategy of microslits makes the sensor exhibit an outstanding ability to resist deformation. Furthermore, Fig. 4d shows the application of the proposed sensor in analyzing the foot-landing action in a gait cycle. This action can be divided into four stages: heel landing, full foot landing, forefoot landing, and toe landing. In this experiment, the sensors were installed on the PI substrate to make a smart insole, as shown in Fig. 4e. The detailed pressure distributions and the output signal of the proposed sensors are presented in Fig. 4f, which shows that the proposed sensor could accurately distinguish the motion states of the four stages in Fig. 4d. Figure S17 presents the stabilized output signals of each sensor during 96 normal walking gait cycles. One of the gait cycle signals (from 72.0 s to 73.7 s) was selected and analyzed, as plotted in Fig. 4g, where the four blue dashed lines represent the four stances shown in Fig. 4d. The rapid response and recovery capabilities of the proposed sensors allowed for sequential pressure increases from sensor 6 to sensor 1 (except for sensor 4), which is highlighted by the orange arrow in Fig. 4g. This result was consistent with the motion evolution from the heel contact to the toes being lifted off the ground. By using the pressure measurements at the medial cuneiform, pronation and supination could be diagnosed at an early stage. As shown in Fig. 4g, the signal of sensor 4 changed slightly during the considered action, indicating a neutral gait and no abnormalities. This result further confirmed that the proposed periodic microslits in the sensing film could help to obtain distinct response signals accurately for different parts of the soles, thus realizing gait health monitoring. Importantly, vehicle load detection could also be achieved using the sensor devices with the designed microslits, as illustrated in Fig. 4h, i. The distinct average pressures of 466.10 kPa, 488.21 kPa, and 516.41 kPa were utilized to determine the weights of vehicles for varying numbers of passengers. The corresponding CA/TA ratios for these pressures are 30.44%, 31.46%, and 32.74%, respectively, and were calculated based on the middle inset of Fig. 1f. The results indicated that the proposed sensor could still maintain stable functionality even under pressures exceeding 400 kPa (i.e., CA/TA of 26.7%) in practical applications. According to the results, the microslits in a sensing film are beneficial to holding the structural deformation under ultrahigh pressure, achieving robust pressure tolerance. The abovementioned results demonstrate that the proposed microslits fabricated in the sensing film could be a viable strategy for realizing accurate, sensitive, and real-time monitoring of the ambient atmosphere, human health diagnosis of diseases, and loading detection.

## Conclusion

This paper presents the successful preparation of a piezoresistive sensing film made of MW-CNT/PDMS with periodic microslits. Based on the synergistic effect of the microslits and submicrostructures in sensing performance, the fabricated sensor exhibits remarkable properties, such as an ultrahigh stress tolerance achieved under a pressure of 400 kPa and a theoretical maximum of 2.477 MPa, as well as a good sensitivity of 18.092 kPa^−1^ in the pressure range of 0–10 kPa. Simulation results and in situ observations demonstrate that the proposed sensing film can effectively accommodate deformation and achieve stress relief under applied pressure to widen the detection range. In addition, the film displacement expands uniformly in all directions, and additional contacts are formed between adjacent cells, thereby enhancing the sensitivity. The outstanding stability of the proposed sensor is demonstrated and validated using experiments with more than 50,000 load/unload cycles. The effectiveness of the proposed sensor in practical applications is therefore experimentally validated in testing that evaluates the proposed sensor’s performance for detecting weak wind signals, supporting health monitoring, and detecting vehicle load. Finally, the findings of this study offer valuable insights into the design and fabrication of flexible devices with ultrahigh stress tolerance and thus contribute to the realization of ubiquitous and diversified pressure monitoring in the future.

## Experimental section

### Materials and reagents

PDMS and its curing agent were purchased from Dow Corning (Sylgard 184), America. MW-CNT with a purity of 95%, a length of 10 μm–30 μm, and a diameter of 10–20 nm were purchased from Jiangsu XFNano Materials Tech. Co., Ltd., China. Terpineol was purchased from Wuxi Yatai United Chemical Co., Ltd., China. The PI film with different thicknesses was purchased from Shanghai Texiang Electrical Insulation Materials Co., Ltd., China.

### Preparation of sensing films with periodic microslit structures

The sensing films used in this study were prepared using screen printing technology, as illustrated in Fig. S1. The fabrication process involved mechanically mixing the MW-CNT and PDMS to create a mixture. The weight ratio of the MW-CNT to the PDMS was varied to examine its influence on the proposed sensor’s performance. Particularly, for the mixtures with MW-CNT, PDMS weight ratios of 0.05:1, 0.1:1, 0.15:1, 0.2:1, 0.25:1, and 0.3:1 were prepared. In the hybrid slurry, MW-CNT were selected as a conductive component because of the high aspect ratio and high conductivity, which made it easier to form conductive pathways in the polymer matrix. Then, terpineol was added to adjust the viscosity of the composite to make it suitable for screen printing. Subsequently, the sensing film layer patterns (1 cm × 1 cm) were screen printed. The film was heated to 120 °C to evaporate the excess solvent. The different microslit structures were shaped by the synergy of the mesh blocking and the flow leveling of the mixture with different weight ratios.

### Characterization

The microstructure and surface topography of all the sensing films were analyzed using field emission SEM (SU-8010, Hitachi, Ltd., Japan). The changes in the colloidal chemical structure before and after the addition of carbon nanotubes were measured using Raman spectroscopy. Specifically, a LabRAM HR evolution instrument from Horiba Scientific, Japan, was employed for this analysis. Furthermore, the changes in the contact area in the sensing were observed using an inverted metallographic microscope (model IE500M) from Sunny Optical Technology (Group) Co., Ltd., China.

### Mechanical and electrical measurements

To measure the currents of the sensors, a bias voltage of 0.1 V was applied, and the resulting current was measured using a source measure unit (2450, Keithley Instruments, USA). The sensitivity curve of the sensor was obtained using a compression loading machine (PT-990T, ChemInstruments International Instruments, China). This machine was used to apply pressure to the sensor and record the corresponding current changes. The specific test methods and schematics are displayed in Fig. S18. To facilitate signal acquisition, the circuit shown in Fig. S10b was employed. This circuit was designed to convert the resistance of the sensor to a voltage in the sensor array and application tests. To investigate and evaluate the response time and stability of the proposed sensor under cyclic loads, the experimental setup presented in Fig. S19 was utilized. The setup could apply dynamic pressure to the sensor, accurately simulating real-world conditions.

### Supplementary information

Supporting Information

A visual representation of the evolution of the contact area with applied pressure

Non-contact pressure sensing applications where continuous tiny airflow is continuously applied to the sensor at an angle of 165°

Non-contact pressure sensing applications where continuous tiny airflow is continuously applied to the sensor at an angle of 90°
