# Supplementary material for: Flexible pressure sensors with ultrahigh stress tolerance enabled by periodic microslits
Source: Microsyst Nanoeng. 2024 Feb 8;10:24. doi: 10.1038/s41378-023-00639-4 (PMC10853210; doi:10.1038/s41378-023-00639-4)
Supplement: Supplementary file 1 — Supporting Information [file 41378_2023_639_MOESM1_ESM.docx]

Supporting Information

**Flexible Pressure Sensor with Ultrahigh Stress Tolerance Enabled by Periodic Microslits**

Song Wang, Chenying Wang, * Yifan Zhao, * Yujing Zhang, Yaxin Zhang, Xiangyue Xu, Qijing Lin, Kai Yao, Yuheng Wang, Feng Han, Yu Sun, and Zhuangde Jiang

S. Wang, Yu. Zhang, Ya. Zhang, X. Xu, Z. Jiang

State Key Laboratory for Manufacturing Systems Engineering, International Joint Laboratory for Micro/Nano Manufacturing and Measurement Technologies, School of Mechanical Engineering, Xi'an Jiaotong University, Xi'an, China

C. Wang, Y. Zhao, Q. Lin, F. Han
State Key Laboratory for Manufacturing Systems Engineering, International Joint Laboratory for Micro/Nano Manufacturing and Measurement Technologies, School of Instrument Science and Technology, Xi'an Jiaotong University, Xi'an, China

E-mail: wangchenying@mail.xjtu.edu.cn, zhaoyifan100@mail.xjtu.edu.cn

K. Yao, Y. Wang

The Faculty of Electrical Engineering and Computer Science, Ningbo University, Ningbo, China

Y. Sun
Department of Mechanical and Industrial Engineering, University of Toronto, Toronto, Canada

**The reason for the formation of morphologies with different MW-CNT/PDMS weight ratios.**

The formation process of different microslits structures is affected by both the fluidity of the MW-CNT/PDMS complex and the mold action of the screen printing plate. When the ratio is low, the flowability of the complex is fine, and at the end of the screen print, the complex will quickly level out, filling in the microslits created by the action of the screen mold. As the ratio of MW-CNTs increases, the fluidity of the complex weakens, which allows for enhanced mold action of the screen (as shown in the inset of the screen printing step in **Figure S1**), ultimately generating microslits morphology. The larger the ratio of MW-CNT, the wider and more irregularly shaped the resulting microslits are, as demonstrated by the morphology of films with the ratios of 0.2:1 and 0.3:1.

**
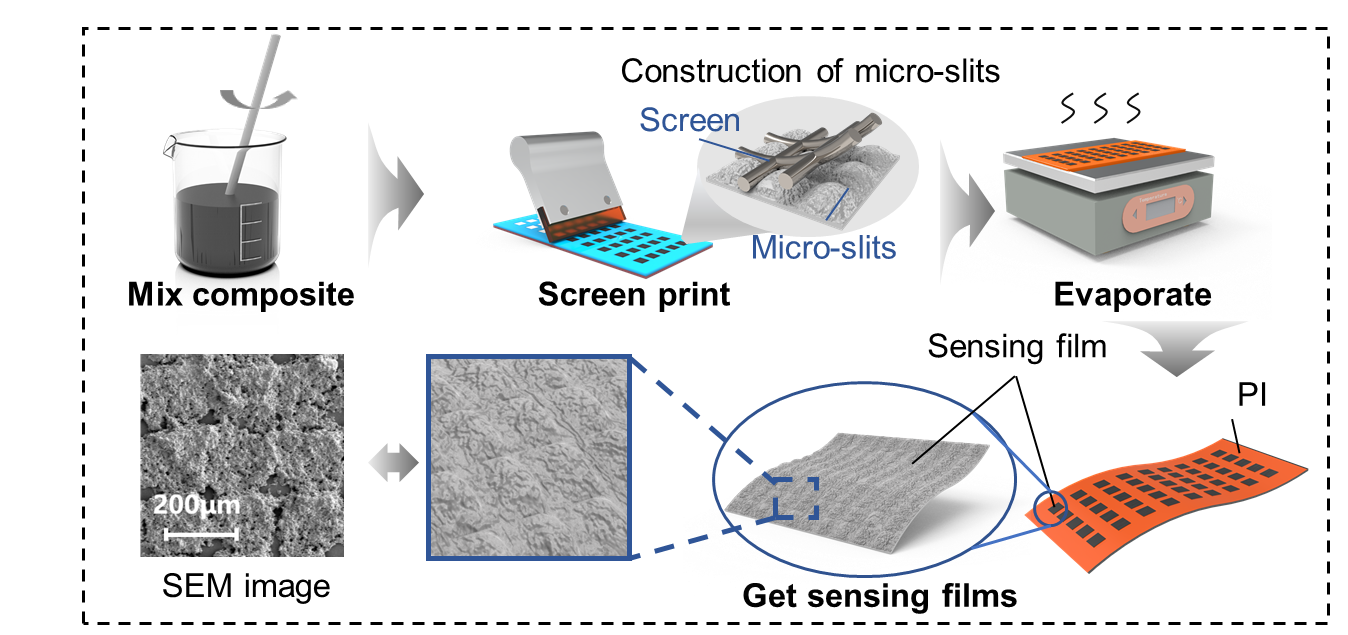
**

**Figure S1.** Schematic of the simple and efficient preparation process of the sensing film with periodic microslits structures.

**Raman characterization analysis of pure PDMS and the PDMS mixed with MW-CNT.**

As shown in **Figure S2**, the Raman spectrum of pure PDMS (black line, labeled PDMS) exhibits six characteristic peaks. The peaks at 489, 614, and 706 cm^-1^ are from the Si-O-Si and Si-C-stretch vibrations of PDMS. The powerful peaks at 1260, 1409, 2906, and 2967 cm^-1^ represent the symmetric bend, asymmetric bend, symmetric stretch, and asymmetric stretch of CH3, respectively. ^[1]^ After the mixing of MW-CNT in PDMS, new distinctive characteristic peaks at 1348, 1581, and 2697 cm^-1^ are observed in the Raman spectrum (red line, labeled MW-CNT/PDMS) in addition to the characteristic peaks that are reflected in PDMS. These peaks correspond to the D-band, G-band, and G' band of MW-CNT, respectively. In contrast, the original peaks at 1260 and 1409 cm^-1^ of PDMS do not appear in the Raman spectrum of the mixture. These phenomena are consistent with the previous reports. ^[2]^

**Figure S2.** Raman spectra of the pure PDMS and the mixture of PDMS and MW-CNTs.


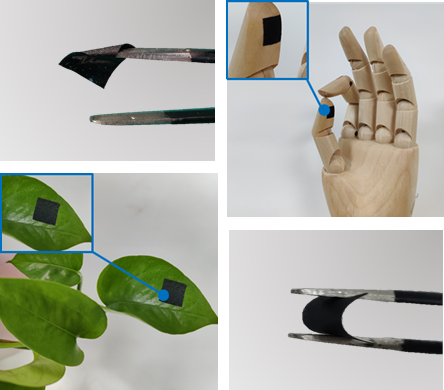


**Figure S3.** An image of the developed sensing film showing its excellent flexibility in arbitrary configurations.


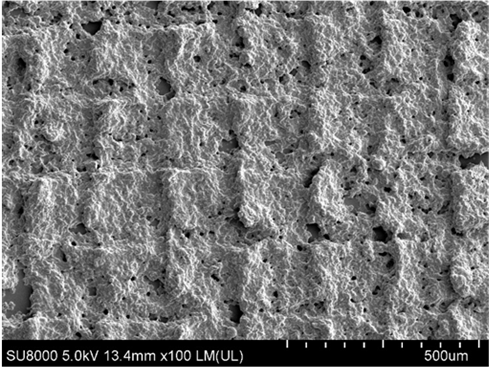


**Figure S4.** SEM image of the NW-CNT/PDMS sensing film with the ratio of 0.1:1.

**Change in the contact area of sensing film under different pressures observed in situ using a microscope.**

The contact process of the microstructure is observed in situ by microscopy to demonstrate the working mechanism of this sensing film (**Figure S5**). The procedure was as follows: firstly, the sensing layer (the sensing film with periodic micro-slits structures) of the sensor was placed upside down on a glass plate; secondly, different weights were applied to the sensing layer; and finally, the observation was made with an inverted metallographic microscope.


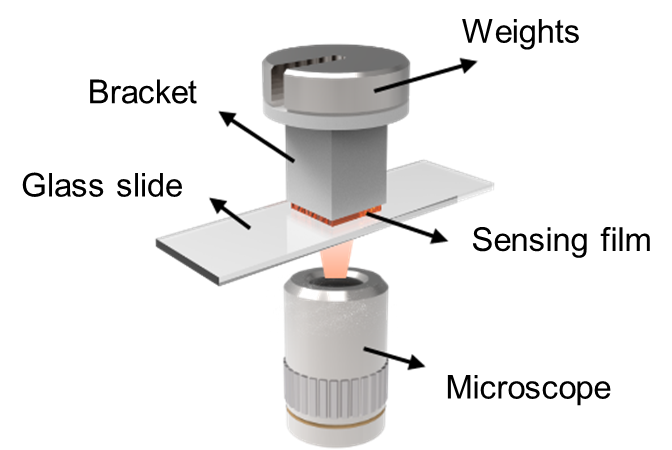


**Figure S5.** Experimental setup for in situ observation of the contact area changes of the sensing film with periodic microslits structures.


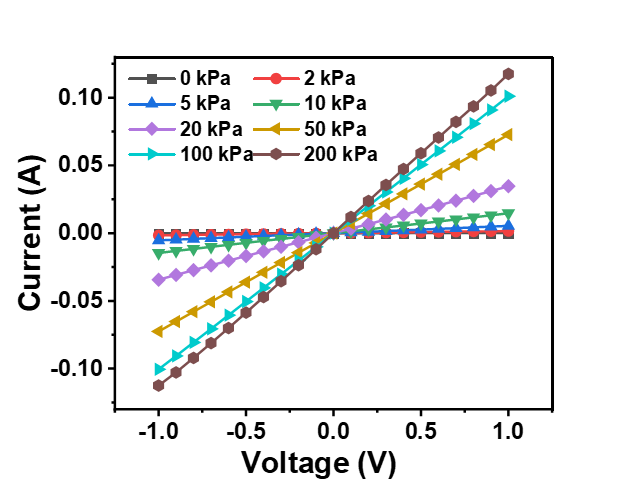


**Figure S6.** Current–voltage (I–V) response curve of the sensor using MW-CNT/PDMS (0.2:1) sensing film when pressure between 0-200 kPa is applied.


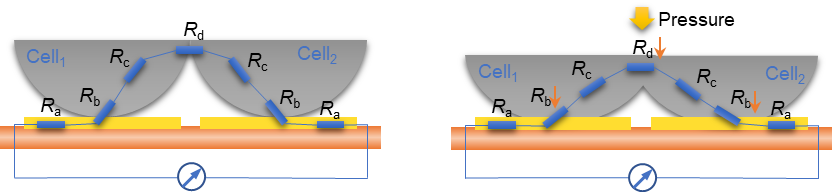


**Figure S7.** Sensing mechanism of the pressure sensor with microslits structures.


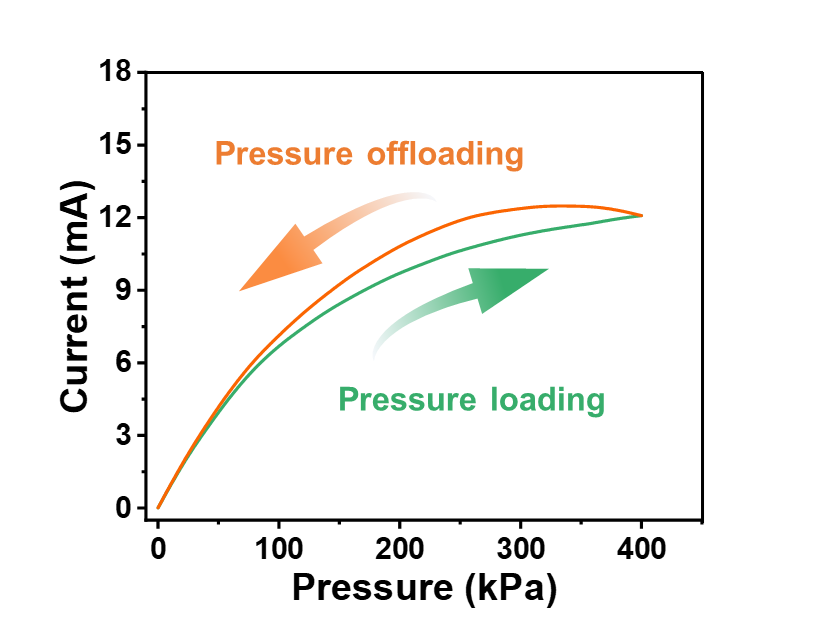


**Figure S8**. Hysteresis curve of the proposed sensor.


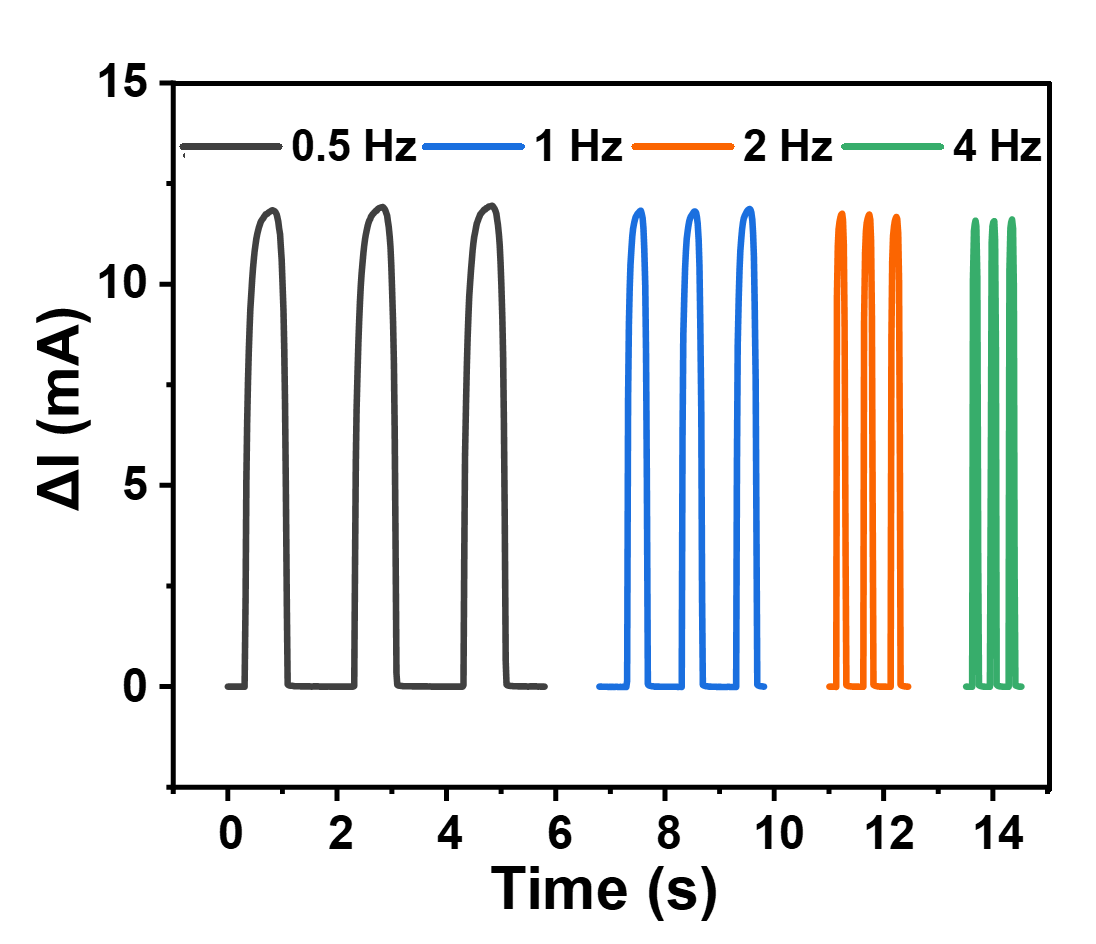


**Figure S9.** Consistent response of the sensor with dynamic pressure loading of 250 kPa at 0.5, 1, 2, and 4 Hz.


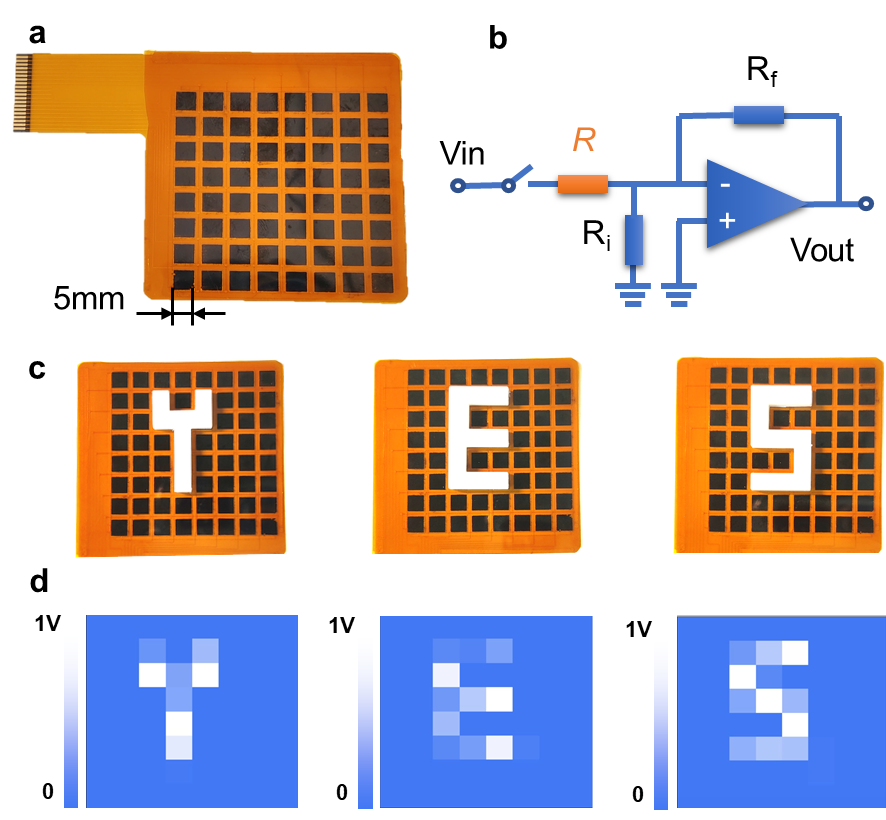


**Figure S10.** (a) Physical photos of the sensor array prepared by one screen printing process, (b) the signal conversion circuit. (c) Schematic of “Y”, “E” and “S” shaped blocks pressed on the prepared 8×8 sensor array. (d) Cloud charts illustration of sensor output with respect to the shape of the input.

**Figure S11.** Effect of temperature variation on the proposed sensor.


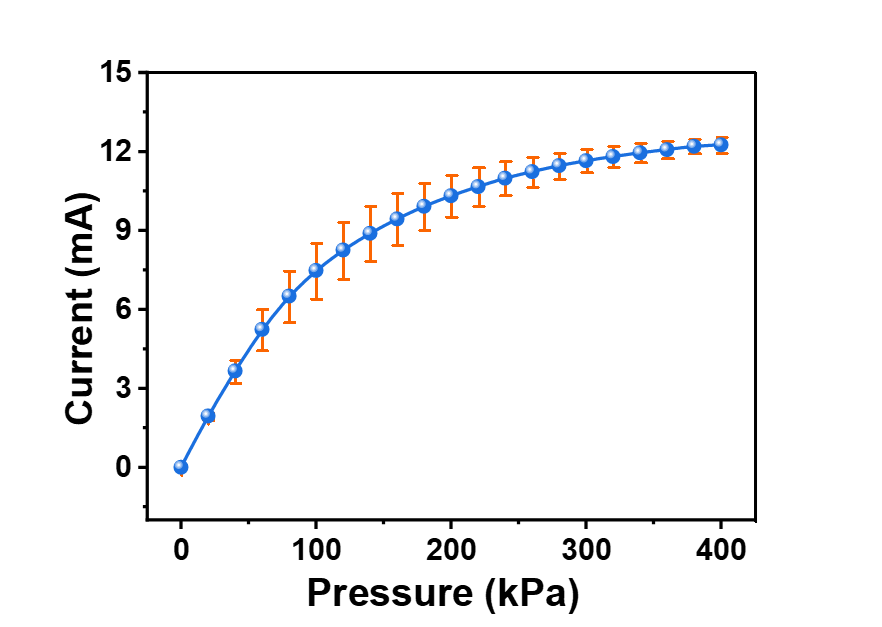


**Figure S12.** The result of different batches of sensors with the MW-CNT/PDMS films (0.2:1).


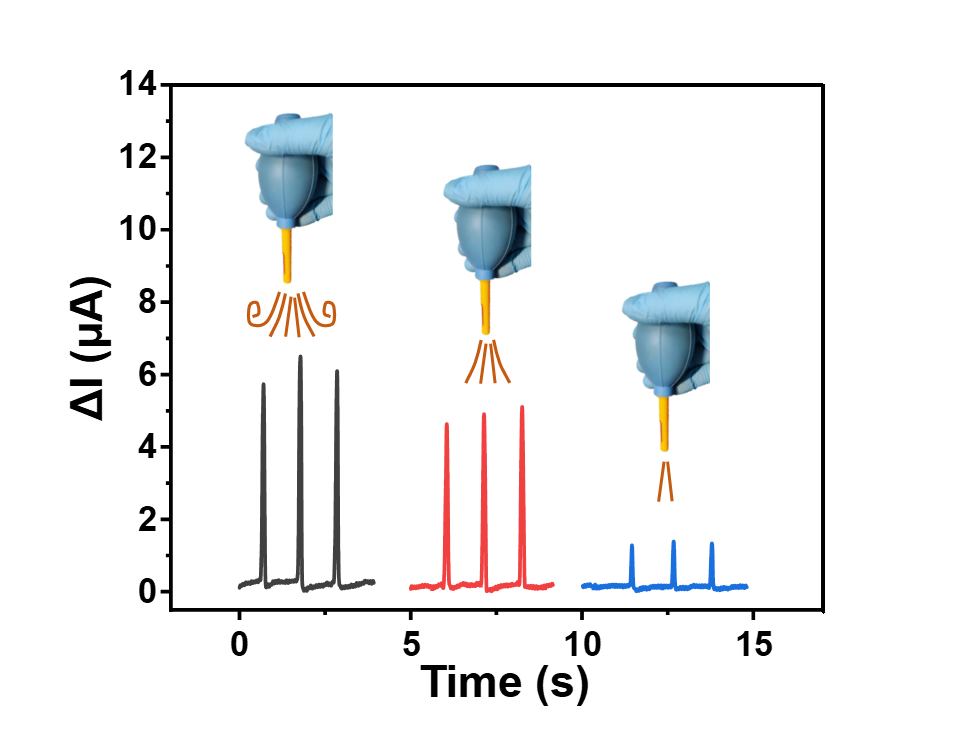


**Figure S13.** Output curves of the proposed sensor at different airflow strengths.

**The potential within the application of sensing films for detecting and recognizing the bending of robot finger joints, grasping objects, and walking postures.**

**Figure S14 (a)** shows the developed sensor mounted on a humanoid mechanical finger; in this application, it achieves bending detection from 0° to 90°. The identification of the elastic modulus of a previously unknown grasped object can assist the image recognition technology in more accurately determining the characteristics of the grasped object. When using the sensor with mechanical jaws, the jaws are used to grip the elastic block (that can be made of different materials of differing elastic moduli), and the elastic modulus of the elastic block can be characterized by evaluating the ratio of the applied force to the deformation of the elastic block when the force is applied; a calculation of this nature is shown in **Figure S14 (b)**. As shown by the red line in **Figure S14 (b)**, the current output of the sensor decreases as the elastic modulus of the object decreases; this indicates that the elastic modulus of the object can be detected through measurement of the change in the current output of the sensor at a given deformation; this increases the capability of the robot to identify previously unknown objects. The excellent agreement shown by the results of six clamping cycles in **Figure S14 (c)** attests to the repeatability of the sensor in practical applications. The developed sensors were also installed on the underside of both the left and right feet of a humanoid robot, and the pressure changes that occurred when the robot walked placing a load alternately on either foot and when moving from standing to sitting position were measured separately. According to the measured voltage curves (**Figure** **S14 (d)** and **S14 (e)**), the contact state between the feet of the robot and the ground can be identified; this information can be used to control the posture of the robot and provide feedback for use in intelligent closed-loop control systems.


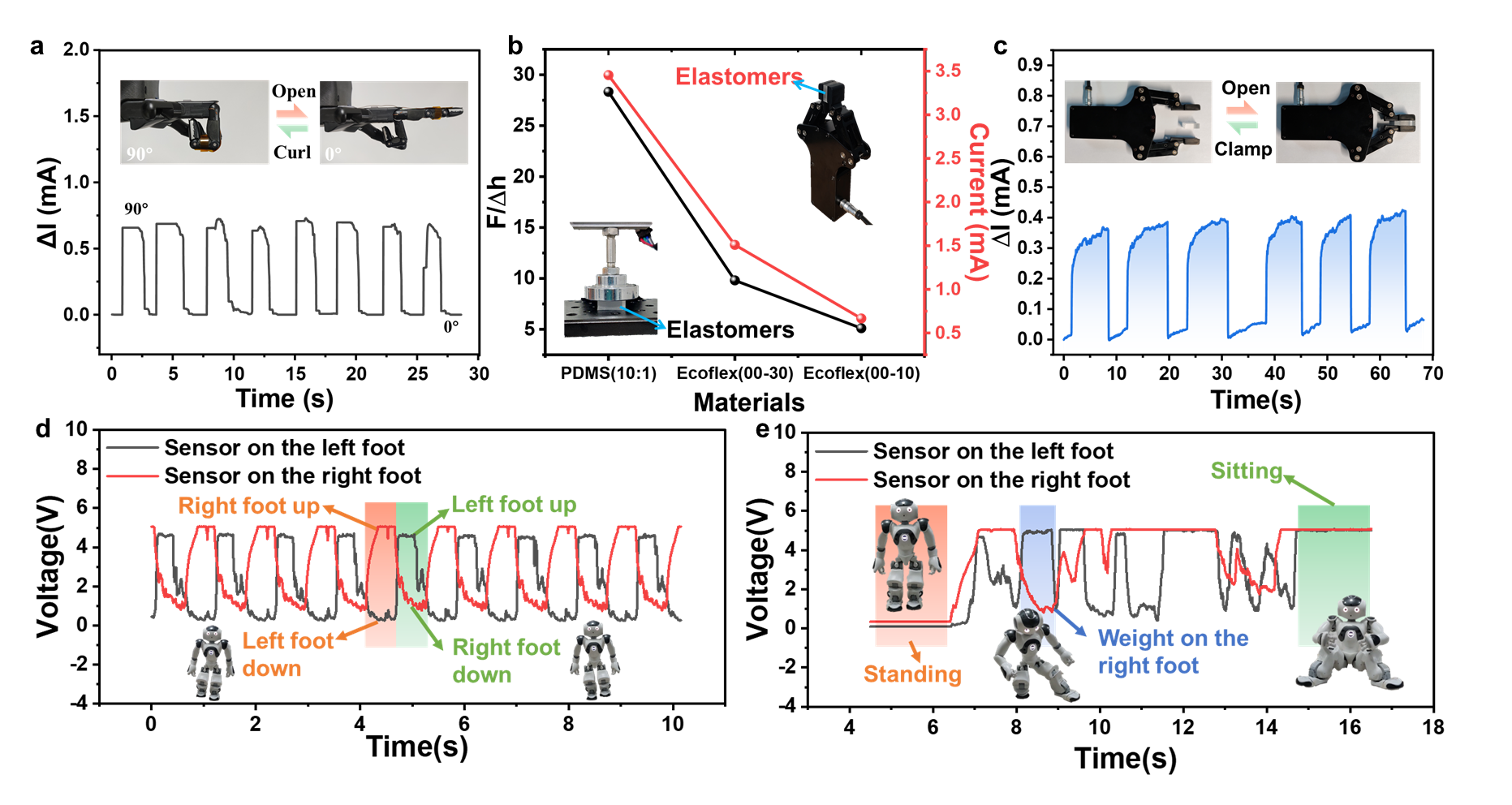


**Figure S14.** Applications of the sensor in the intelligent posture monitoring of humanoid robots and monitoring of plantar pressure distributions in humans. (a) The developed sensor serves as a bend detection sensor to measure the bending of the robotic finger. (b) The current response obtained in a robotic jaw with the developed sensor gripping different elastomers with different moduli of elasticity (red line). The ratio of force to deformation imposed on the different elastomers is used to evaluate the modulus of elasticity of the elastomers (black line). The prepared sensors were also installed on the underside of the left and right feet of a humanoid robot to detect the pressure distribution during (d) walking and (e) transitioning from standing to sitting positions (both feet off the ground). The postures of the robot can be inferred from the sensor readings.


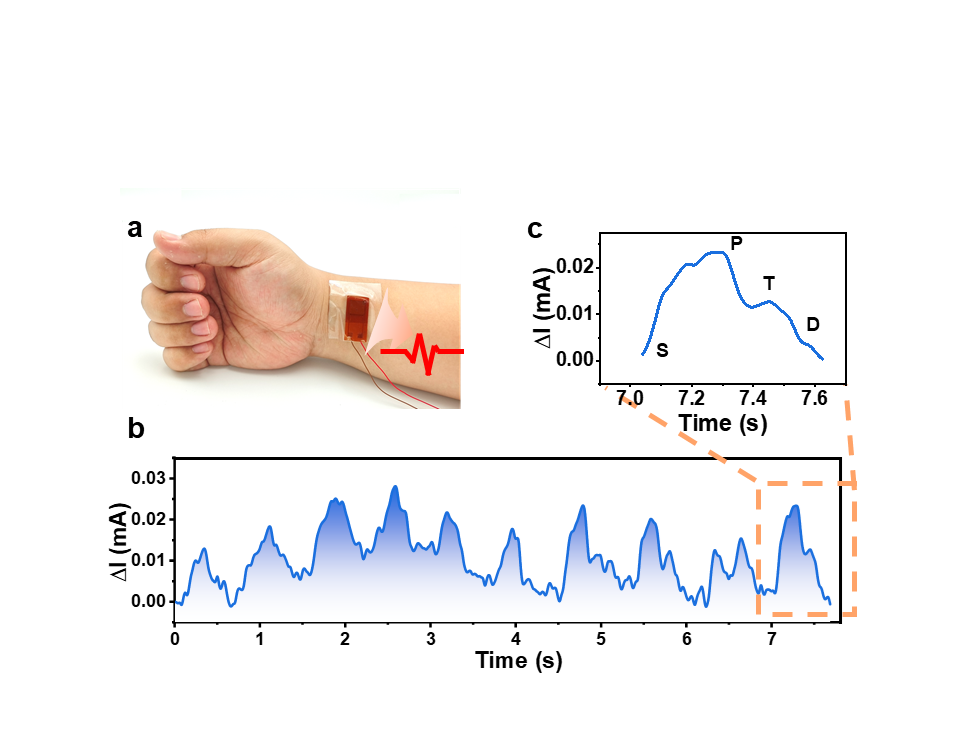


**Figure S15.** The developed sensor is applied with pulse monitoring. (a) The sensor is mounted on the wrist using medical tape. (b) The pulse signal of the tested volunteers. (c) Specific recognition of pulse signature peaks.

**Figure S16.** Performance curves of the proposed sensor before and after 1,000 bending cycles.

**Figure S17.** Output signals of 6 sensors on the insole over 96 normal walking gait cycles.

**Experimental setup for testing piezoresistive characteristics.**

The experimental setup shown in **Figure S18** was adopted to test the output characteristics of the sensor under different pressures. The press with the moving speed of 1 mm/min was used to apply different pressures. A bias voltage of 0.1 V was applied to the prepared sensor using a computer-controlled source measure unit (SMU), and its current was measured simultaneously.


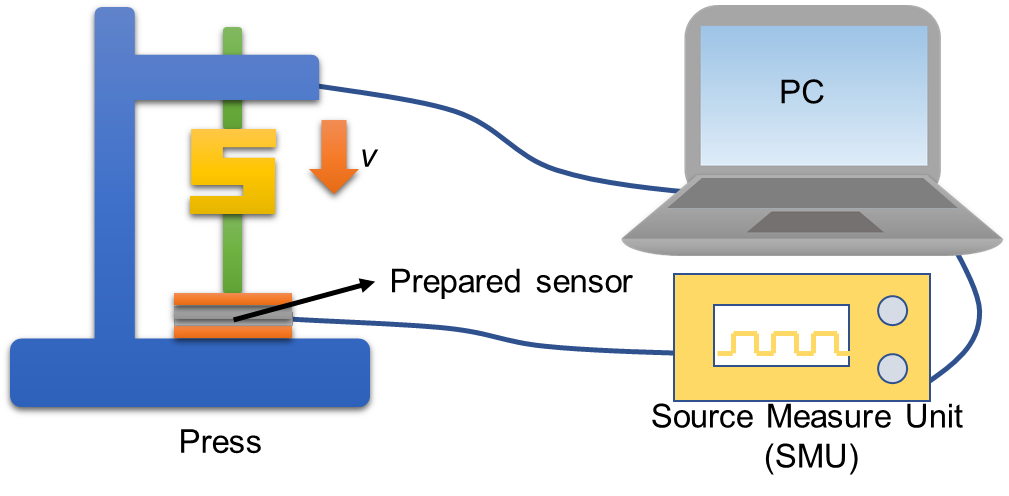


**Figure S18.** The experimental setup when applying different pressures to the prepared sensor.

**The method for applying different types of dynamic pressure to sensors.**

As shown in **Figure S19**, forces of different frequencies and types are obtained by a function signal generator, and then transmitted to a modal shaker by a power amplifier. The mandrel of the modal shaker acts on the prepared sensor with various forces transmitted from the function signal generator. The magnitude of the force is carried out by adjusting the gain of the power amplifier, and the frequency and amplitude of the applied force are obtained from time to time through a commercial sensor mounted on the mandrel.


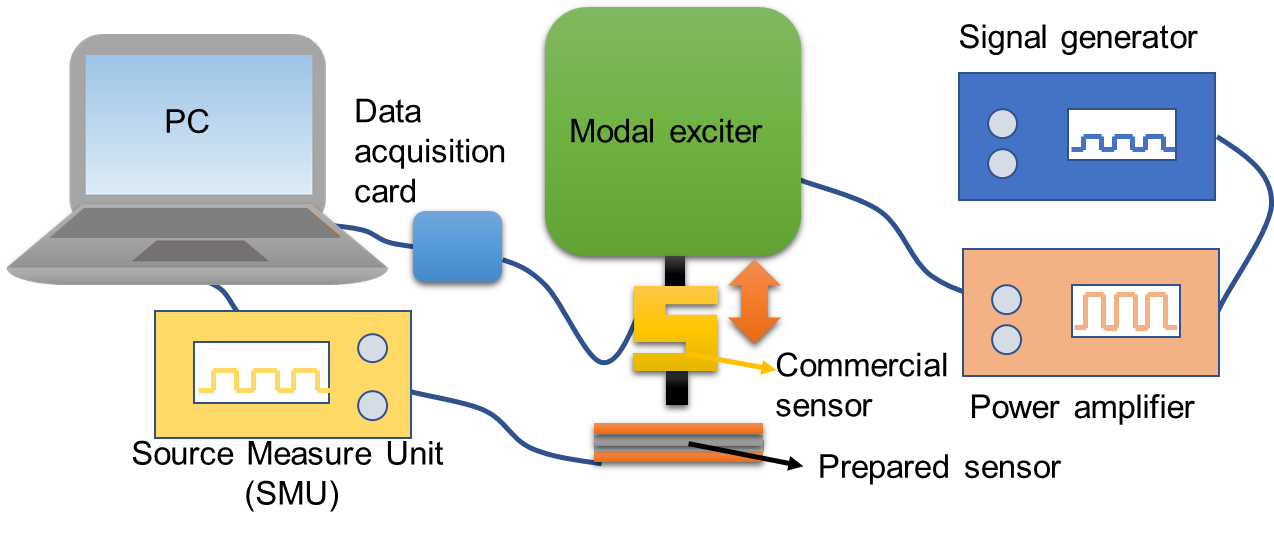


**Figure S19.** The experimental setup when dynamic pressure is applied to the sensor.

**Finite element simulation setup of different microslits structures**

A finite element method was used to simulate the deformation and stresses of three structures subjected to external pressure at different mixing ratios (0.05:1, 0.2:1, and 0.3:1 for MWCNs and PDMS, respectively). The mechanical parameters were set identically for the three structural models. The material was chosen to be PDMS (Young's modulus of 750 kPa, Poisson's ratio of 0.49, and density of 970 kg/m^3^), which is an isotropic linear elastic material. Each microstructure unit is simplified as a hemisphere of 1m radius, and the upper part of the microstructure is divided into three kinds of connected, unconnected and irregular structures. It is assumed that the lower surface is fixed and initially stationary. The same stress of 100 N/m^2^ is applied on the upper surface. At steady state, the distribution of physical parameters reflecting the properties of the structure, such as displacement and stress distribution, can be obtained by the equilibrium equation, compatibility equation, and Hooke’s law.
Equilibrium equation is

$\text{∇⋅}\text{σ}\text{+}\text{F}_{\text{ν}}\text{=}\text{ρ}\frac{\text{∂}^{\text{2}}\text{u}}{\text{∂}\text{t}^{\text{2}}}\text{=0}$;

Compatibility equation is

$\text{ε}\text{ }\text{=}\frac{\text{1}}{\text{2}}\text{[}{(\text{∇}\text{u})}^{\text{T}}\text{+}\text{∇}\text{u}\text{]}$;

Hooke's law is

$\text{σ}\text{ }\text{=D}\text{⋅}\text{ε}$ ($\text{D=}\frac{\text{E}}{\left( \text{1+}\text{v} \right)\left( \text{1-2}\text{v} \right)}$),

where $\sigma$ is the stress, $\text{F}_{\text{ν}}$ is the force per unit volume, $\text{u}$ is the displacement, $\text{ε}$ is the strain, $\text{D}$ is the elastic matrix, $\text{E}$ is the Young's modulus, and $\text{v}$ is the Poisson's coefficient.

References

[1] Cai, D., Neyer, A., Kuckuk, R. & Heise, H. M. Raman, mid-infrared, near-infrared and ultraviolet-visible spectroscopy of PDMS silicone rubber for characterization of polymer optical waveguide materials. *J. Mol. Struct.* 976, 274-281 (2010).

[2] Mu, C. et al. Flexible strain/pressure sensor with good sensitivity and broad detection range by coupling PDMS and carbon nanocapsules. *J. Alloy. Compd.* 918, 165696 (2022).
